# Editorial: NK cell defects: diagnosis and treatment

**DOI:** 10.3389/fimmu.2023.1323793

**Published:** 2023-10-31

**Authors:** Snehal Shabrish, Shanmuganathan Chandrakasan, Manisha Madkaikar

**Affiliations:** ^1^ Translational Research Laboratory, Advanced Centre for Treatment, Research and Education in Cancer, Tata Memorial Centre, Navi Mumbai, India; ^2^ Homi Bhabha National Institute, Mumbai, India; ^3^ Department of Pediatrics, Aflac Cancer and Blood Disorders Center, Children’s Healthcare of Atlanta, Emory University School of Medicine, Atlanta, GA, United States; ^4^ Department of Pediatric Immunology & Leukocyte Biology, Indian Council of Medical Research - National Institute of Immunohaematology, King Edward Memorial (KEM) Hospital, Mumbai, Maharashtra, India

**Keywords:** NK cell, NK cell-related gene signature, Fanconi anemia, familial hemophagocytic lymphohistiocytosis, NK cell degranulation defects, NK cell based therapeutics, NK cell biology

Natural Killer (NK) cells form an integral component of the immune response, especially against virally infected cells and cancer cells (1). They have gained diagnostic and prognostic significance in the last few decades with increasing literature portraying their role in inflammatory disease pathology and therapy (2, 3). However, even though these cells have importance in research set-up, this significance has not yet reached routine clinical set-up. Various inherited genetic defects, pathological conditions, and therapies are known to have immunosuppressive effects on NK cells, causing NK cell defects (4, 5). However, in many of these cases, the mechanism remains unclear. This could be attributed to the lack of studies focusing on NK cell immune biology, the influence of varied pathological conditions on its immune phenotype and functions; and its impact on outcome. In the future, translation of this knowledge from “bench-to-bedside” will definitely contribute to better diagnosis and therapeutics in multiple pathological conditions. Thus, the main focus of this Research Topic was to explore recent advances in understanding NK cell defects, their diagnosis, and their treatment. In total, after being peer-review, 4 manuscripts, composed of 3 research articles and 1 review article from 28 researchers were successfully accepted for publication.

The first two research articles aimed at evaluating the utility of NK cell-related gene signature (NRGs) for predicting the prognosis of head and neck squamous cell carcinoma (HNSCC) and Triple-negative breast cancer (TNBC). For HNSCC and TNBC risk stratification by tumor size, distant metastasis and histological grade alone is not sufficient to predict prognosis, and there is an urgent requirement for more accurate models for predicting prognosis (6, 7). In recent years, NK cell-related genes have gained attention as NK cells play a crucial role in the tumor microenvironment and immune surveillance (8, 9). In both these studies, the authors established the NRGs based on TCGA and GEO databases using univariate and LASSO Cox regression analysis. In the study by Chi et al., 17-NRGs signature and nomograms showed excellent predictive performance. These 17-NRGs identified in the prognostic risk model were closely associated with various immune cells including resting as well as activated NK cells. The NRGs risk model was also found to be associated with clinical characteristics and risk scores of all HNSCC patients in TCGA, indicating its utility in predicting the prognosis of HNSCC patients. The other study by Liu et al., established a prognostic signature for TNBC based on 5 NRGs classifying TNBC patients into high-risk and low-risk groups, with better prognostic outcomes in the latter group. Additionally, the authors analyzed the sensitivity to immunotherapies using the tumor immune dysfunction and exclusion (TIDE) algorithm. The results revealed TIDE scores were higher in the high-risk groups than in low-risk groups, indicating that TBNC patients in the NK cell-related low-risk group may respond better to immunotherapy. Both these studies demonstrate the clinical implications of NRGs for both prognostic and therapeutic assessment of patients. However, they have a limitation of being a retrospective study and require further validation in prospective studies. Nevertheless, studies like these form the base for the future to develop new tools for improving treatment options.

The third original research article is on NK cell degranulation defects in Fanconi anemia (FA) patients. FA is a chromosomal instability disorder with inflammatory pathology of unknown origin. Though various immunological abnormalities have been previously reported in FA patients (10), this study for the first time demonstrates in FA patients NK cell degranulation defect, which is a screening assay for familial hemophagocytic lymphohistiocytosis (FHL). NK cell function studies in 9 diagnosed FA patients, showed normal perforin expression, but defective degranulation pattern and NK cell activity. Genetic analysis in these patients revealed mutations in FA-related genes, while no mutation was detected in HLH-related genes indicating that the defect in the degranulation defect in FA patients is not a result of a defect in HLH-related proteins. Further, based on the therapeutic response assessed in these patients, the authors emphasize the need for early suspicion, evaluation for HLH, and prompt initiation of immunosuppressive therapy, especially in a setting of rapidly deteriorating cytopenia in patients with FA. Taken together, this study highlights NK cell degranulation defects, hypercytokinemia, and susceptibility to developing HLH as one of the important mechanisms for cytopenia in FA. The author also proposes that FA genes be included in the list of genes for familial HLH.

The fourth article is a review of the implications of NK cell defects in acute myeloid leukemia. Anti-leukemic properties of NK cells have been explored in the past few years and different NK cell therapies have been implicated in treating AML (11), however, the data available is still very scarce and varied, hindering the clinical utility of these therapies. This review article by D’Silva et al. compiles these findings and gives an overview of NK cell defects in AML progression including defects in number, NK cell receptor (NCR) expression, and maturation pattern. The authors then discuss the therapeutic options for AML with a prime focus on NK cell-based therapies targeting NKG2D-NKG2DL, adoptive NK cell therapy, monoclonal antibody therapy, NK CAR therapy, NK cell engager therapeutics consisting of bi-specific killer engagers (BiKEs) and tri-specific killer engagers (TriKEs), and immune checkpoint blockade therapies. This review also collates the results of various preclinical and clinical trials of these NK cell-based therapies. The authors conclude this review by stating that though multiple studies prove the dominance of NK cell therapies over other cellular therapies, these studies are still in preliminary stages. More extensive studies are required on NK cell expansion, cryopreservation, infusion protocols, and safety for utilizing these therapies in a clinical setting.

Collectively, the articles in this Research Topic highlight the urge for more comprehensive studies for an in-depth understanding of NK cell biology, as it will have diagnostic, prognostic as well as therapeutic implications.

## Author contributions

SS: Conceptualization, Writing – original draft, Writing – review & editing. SC: Writing – original draft, Writing – review & editing. MM: Writing – original draft, Writing – review & editing.
